# Supplementary material for: Effectiveness of Nintendo Wii Fit© for Physical Therapy in Patients with Multiple Sclerosis: A Systematic Review and Meta-Analysis of Randomized Controlled Trials
Source: J Pers Med. 2024 Aug 24;14(9):896. doi: 10.3390/jpm14090896 (PMC11433451; doi:10.3390/jpm14090896)
Supplement: Supplementary file 1 [file jpm-14-00896-s001.zip › Table S1. Literature Search.pdf]

Table S1. Literature search with the search strategy and results of each database.

Database	Search strategy	Results (number of articles)
PubMed	("Multiple sclerosis" OR “Disseminated sclerosis”) AND ("Nintendo Wii" OR "Wii" OR “Wii Fit" OR "Wii Balance Board" OR "exergaming")	57
CENTRAL		59
CINAHL		26
Scopus		74
WoS		83
Medline		56
PEDro	Multiple sclerosis AND Wii	12
	Multiple sclerosis AND Exergaming	9

CENTRAL: Cochrane Central Register of Controlled Trials; CINAHL: Cumulative Index to Nursing & Allied Health Literature; PEDro: Physiotherapy Evidence Database; WoS: Web of Science.
